# Control of neurite growth and guidance by an inhibitory cell-body signal

**DOI:** 10.1371/journal.pcbi.1006218

**Published:** 2018-06-21

**Authors:** Brendan A. Bicknell, Zac Pujic, Peter Dayan, Geoffrey J. Goodhill

**Affiliations:** 1 Queensland Brain Institute, University of Queensland, St Lucia, Queensland, Australia; 2 School of Mathematics and Physics, University of Queensland, St Lucia, Queensland, Australia; 3 Gatsby Computational Neuroscience Unit, University College London, London, United Kingdom; Oxford, UNITED KINGDOM

## Abstract

The development of a functional nervous system requires tight control of neurite growth and guidance by extracellular chemical cues. Neurite growth is astonishingly sensitive to shallow concentration gradients, but a widely observed feature of both growth and guidance regulation, with important consequences for development and regeneration, is that both are only elicited over the same relatively narrow range of concentrations. Here we show that all these phenomena can be explained within one theoretical framework. We first test long-standing explanations for the suppression of the trophic effects of nerve growth factor at high concentrations, and find they are contradicted by experiment. Instead we propose a new hypothesis involving inhibitory signalling among the cell bodies, and then extend this hypothesis to show how both growth and guidance can be understood in terms of a common underlying signalling mechanism. This new model for the first time unifies several key features of neurite growth regulation, quantitatively explains many aspects of experimental data, and makes new predictions about unknown details of developmental signalling.

## Author summary

For the brain to become wired up during development, growing nerve fibres use molecular guidance factors to navigate over long distances and find their appropriate targets. However, the ability of nerve fibres to do this is severely limited by the loss of both growth and guidance as the concentration of guidance factors increases—a phenomenon that has never been fully explained. We propose a mathematical model that couples growth and guidance at the level of signal transduction, and show that it can, for the first time, quantitatively explain the largest current dataset of precisely controlled measurements. This finding impacts on understanding of both the causes of neurodevelopmental disorders, and repair after brain or spinal injury.

## Introduction

The development of the nervous system requires the precise wiring of billions of cells. To achieve this considerable feat, growing axons navigate over long distances to reach their synaptic targets, and hence establish appropriate patterns of connectivity. Dysregulation of this process contributes to developmental and neurological disorders [1, 2], and the inability to recapitulate early growth events hinders nerve injury repair [3].

One major regulator of axonal growth (trophism) and guidance (tropism) is signalling by extracellular chemical cues. A large number of these are known, with nerve growth factor (NGF) being perhaps the best studied [4–8]. The effects of NGF on growth and guidance are exerted via tight control of signalling along the axon for extension and turning, and at the cell-body to coordinate synthesis and supply of raw materials. As might be expected, neither growth nor guidance occurs at very low concentrations. More puzzling is that they are also both inhibited at higher concentrations, exhibiting a biphasic dose response that peaks in an intermediate concentration regime [9–18]. Such tight constraints on both growth and guidance are important since they increase the challenge for therapeutically effective interventions.

Existing theories of the biphasic effects of NGF on growth depend on mechanisms intrinsic to single cells [16, 19–21], or a collective effect in which growth within aggregates of cells is hindered by increased fasciculation (the grouping of neurites into bundled fibres) [15]. However, these theories remain purely qualitative and lack thorough experimental tests. Two fundamental mechanisms underlie guidance: turning and differential growth. Turning largely occurs for steep gradients. The decline in its sensitivity with overall concentration can therefore be explained by saturation of finite numbers of receptors [18]. However, for shallow gradients, (for NGF, < 1% concentration change per 10 μm) guidance is remarkably sensitive, and depends on preferential growth towards higher concentrations [17, 22]. In this paradigm of chemotactic response, neurites do not exhibit biased turning, but grow more quickly or more slowly when extending up or down a gradient, respectively. The biphasic effects of NGF on guidance by differential growth therefore inherit the puzzle of those on growth in general. Intriguingly, however, even in the decreasing portion of the dose response curve for growth, where lower concentrations should generate more growth than higher concentrations, differential growth remains biased up the gradient [22]. Thus, guidance involves a true detection of the gradient, yet as it is also inextricably linked to growth, this tropic response cannot be fully understood in isolation.

To address these issues we first test previous proposals for NGF growth inhibition at high concentrations, and find they do not explain the biphasic response of dorsal root ganglia (DRG) explants in collagen gels. Second, inspired by a reanalysis of the extensive shallow gradient data set of ref. [18], we propose a novel signal transduction mechanism which resolves the apparent contradictions introduced above. In this, the growth at the neurite tip is promoted by the local concentration of NGF, but, critically, is also inhibited by a somatically-computed signal that results from NGF-dependent signalling among the collection of cell bodies. Anterograde transport from the soma modulates growth by supply of signalling components, whereas retrograde transport from the tip provides the soma with information about the distal concentration. The inhibition implements the decrease in growth at high concentrations, and provides the normalisation that allows differential growth to be a suitably sensitive guidance mechanism. The model represents a new signalling paradigm for understanding nervous system development and repair, and makes testable predictions applicable to NGF and other growth and guidance cues.

## Results

### Growth inhibition at high concentration is neither single-cell intrinsic, nor due to fasciculation

There are two main hypotheses as to how high concentrations of NGF inhibit growth. Based on experiments on chick thoracic DRGs, ref. [15] proposed that an NGF-dependent increase in neurite fasciculation hinders growth from aggregates of cells, and reported no effect in single cells. Conversely, others have proposed that inhibition acts at the single-cell level, such as by TrkA receptor saturation or downregulation [16, 19, 21] or signalling via the low affinity receptor p75 [20]. To test these competing theories, we measured the NGF response of early postnatal rat DRG explants and dissociated cells, grown together at low density in collagen gels for 48 h.

Previous studies have quantified explant outgrowth by manual measurements of radial extension [15, 16, 19], semi-automated measurement of area and density [17, 18, 22, 23], and least-squares fitting of ellipses to explant shape [24]. However, manual measurements become impractical for large data sets, and previous semi-automated methods provide only coarse descriptions of collective outgrowth that are difficult to interpret at a lower level. To gain a more detailed understanding of growth patterns, we instead used a Fourier decomposition that is capable, in principle, of capturing arbitrary patterns of neurite extension, and is directly related to specific features of the response. Briefly, we fitted boundary curves to the central cell-body region of the explant and to the outer limit of neurite outgrowth, and parameterised the distance between the curves with an angular variable. The Fourier coefficients of this radial outgrowth function quantified the average radial outgrowth *a*_0_ (average distance between explant body and limit of neurite extension), outgrowth bias in orthogonal image axes *a*_1_, *b*_1_, and other higher-order features (Methods).

**Fig 1:**
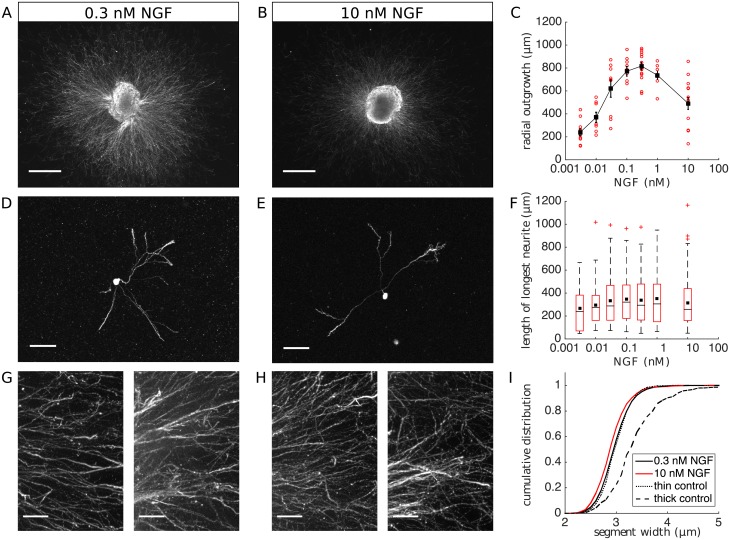
NGF dependence of neurite growth and fasciculation. DRG explants and dissociated cells grown in collagen for 48 h with various NGF concentrations were stained with TuJ1 for neurite visualisation. (A,B) Representative examples of explants at 0.3 nM and 10 nM demonstrate the reduction in outgrowth at high NGF concentrations (scale bars 500 μm). (C) Quantification of average explant outgrowth shows a biphasic NGF dependance. Red markers correspond to individual explants, error bars are SEM. (D,E) Representative examples of dissociated cells at 0.3 nM and 10 nM demonstrate the absence of growth inhibition (scale bars 100 μm). (F) Quantification of dissociated-cell neurite lengths. Black lines are medians, black squares are means, and red crosses are outliers from 20 − 156 cells per condition. (G,H) Representative images of neurites extending from explants in 0.3 nM and 10 nM NGF concentrations at higher magnification (scale bars 50 μm). (I) A comparison of neurite bundle widths shows no increase in fasciculation at 10 nM NGF. Our method of image analysis easily detects differences in control sample patches of thin and thick bundles.

The average radial outgrowth of the explants exhibited the expected biphasic response curve (Fig 1A–1C). Explant outgrowth peaked at 0.3nM NGF concentration (mean 817 μm), and was comparatively reduced (mean 491 μm) at 10 nM (*p* = 1 × 10^−4^, Mann-Whitney U-test for difference between 0.3 nM and 10 nM conditions, *n* = 15 explants per condition, 8 animals from 4 separate experiments). By contrast, recording the length of the longest neurite of each dissociated cell (Fig 1D–1F), we observed no evidence of growth inhibition at high NGF concentrations (*p* = 0.35, Mann-Whitney U-test for difference between 0.3 nM and 10 nM conditions, *n* = 126 and *n* = 156 cells respectively). Comparing dissociated cells in the 0.1 nM condition, which exhibited the highest median neurite length, with the 10 nM condition gave a similar result (*p* = 0.2, Mann-Whitney U-test, *n* = 71 cells for 0.1 nM). The results of both comparisons were robust to the removal of outliers (defined as values lying more than 1.5 times the interquartile range above the third quartile; Fig 1F). To test whether the lack of observed effect in dissociated cells may be due to a growth latency caused by the dissociation procedure, we repeated the experiment with an extended period of 96 h total growth. Consistent with the 48 h results, we observed a pronounced difference in explant outgrowth after 96 h, but no detectable difference in dissociated-cell neurite length distributions (S1 Fig). Thus, inhibition of growth at high NGF concentrations is a property of intact ganglia, and not of isolated single cells.

To assess the effect of NGF on fasciculation (cf. ref [15]), we acquired higher resolution images of explants in the 0.3 nM and 10 nM conditions, and performed an automated image analysis to compute distributions of neurite bundle widths (Fig 1G–1I). As a positive control, we tested the ability of our method to discriminate distributions from sample patches containing mainly thick or thin bundles as judged by eye (taken from both NGF conditions, see S2 Fig for examples). The difference between control samples, corresponding to a 1 − 2 μm increase in bundle widths, was easily detected (*p* = 5 × 10^−30^, one-tailed Mann-Whitney U-test, *n* = 353 segments and *n* = 414 segments for thick and thin respectively). Applying the automated analysis to the test conditions, we found no detectable increase in bundle widths at 10 nM compared with 0.3 nM (*p* = 1, one-tailed Mann-Whitney U-test, *n* = 1687 segments and *n* = 2546 segments for 10 nM and 0.3 nM respectively, from 8 explants each). Thus, in our system, increased fasciculation does not explain the biphasic NGF response. This suggests the correlation between fasciculation and growth inhibition observed by ref. [15] is not a causal relationship, nor is it a general property of NGF-dependent growth regulation.

As the results of our experiments contradict previous suggestions [15, 16, 19–21], we propose an alternate mechanism for neurite growth regulation. A fundamental difference between an intact ganglion and a single dissociated cell is the central mass of neuronal cell bodies and support cells that comprise the ganglion body. This suggests the possibility that an NGF-dependent signal within the collection of cell bodies plays an inhibitory role in ganglion outgrowth. This may be mediated, for instance, by cell-cell interactions in which a paracrine factor is secreted from the cell bodies and inhibits neighbouring cells (as shown for regulation of cell survival [25]). Another possibility is that satellite glial cells within the ganglion, which also carry NGF receptors [26], communicate an inhibitory signal to the neural cell bodies that they ensheathe. By analogy with other systems [27, 28], we further propose that this inhibitory mechanism permits sensitive gradient detection, and may thus explain the guidance by differential growth observed by ref. [22]—there termed guidance by growth rate modulation. We develop this hypothesis with a mathematical model, and thus build a quantitative and predictive description of inhibitory growth and guidance signalling.

### Biased outgrowth in shallow gradients implies remarkable sensitivity

**Fig 2:**
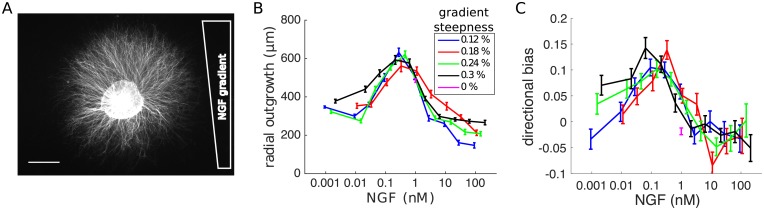
Analysis of NGF gradient data set. (A) Example image from the data set of ref. [18], which documents the total growth of DRG explants after 48 h in shallow NGF gradients. Experimental conditions were varied over gradient steepnesses 0 − 0.3% concentration change per 10 μm, and background concentrations of ≈ 0.001 − 100 nM (scale bar 500 μm). (B) Average radial outgrowth, quantified by Fourier coefficient *a*_0_, has a biphasic dependence on background NGF concentration, and is largely independent of gradient steepness. (C) Directional bias up the gradient, quantified by normalised coefficient *b*_1_/*a*_0_, was observed over background concentrations 0.01 − 1 nM, implying a high sensitivity to NGF.

To constrain the model, we first applied our explant image analysis to the NGF gradient data set of ref. [18], which documents the growth of DRG explants after 48 h in very shallow NGF gradients in collagen gels. The data set comprises 3460 images of explants in which the ganglion body region had been manually segmented from the neurite region. Gradient parameters in the experiments varied between 0 − 0.3% concentration change per 10 μm, and background concentrations ≈ 0.001 − 100 nM, making it the most extensive record of NGF growth regulation yet compiled. An example image of an explant is shown in Fig 2A, here displaying a pronounced bias in neurite outgrowth in the direction of increasing NGF concentration.

Applying the image analysis to the data, only two Fourier coefficients, *a*_0_ and *b*_1_, varied systematically with the gradient parameters. We thus quantified the growth response by the average radial outgrowth *a*_0_, and directional bias *b*_1_/*a*_0_, which gives the fractional increase in neurite extension on the up-gradient side of the explant, relative to the average (or fractional decrease on the down-gradient side). Explants with less than 100 μm average radial outgrowth (125/3460; 4%) were excluded from the analysis of directional bias. Consistent with the results of ref. [18], and Fig 1C, the average radial outgrowth exhibited a biphasic NGF dependence, with a peak at 0.3 nM and inhibition at higher concentrations (Fig 2B). Outgrowth was biased in the direction of the gradient for background concentrations between 0.01 − 1 nM (Fig 2C, S1 Table). By contrast, analysing the explant-body boundary curves alone, we found that the average explant body boundary was well-approximated by a circle of radius *R*_*E*_ = 300 μm. We found no correlations in shape properties within or between the outgrowth and explant body regions (S2 Table).

The peak directional bias was observed at ≈ 0.1 nM in a 0.3% gradient, in which neurites facing directly up the gradient extended ≈ 15% further than the average over the explant. With these gradient parameters and the measurements in Fig 2B, this implies a ≈ 100 μm increase in growth has resulted from a maximum concentration difference of only 0.03 nM across the full length of the neurites. Observed over two orders of magnitude of background concentrations, this remarkable response involves a tropic growth modulation [22], and places a strong constraint on any proposed mechanism.

### A mathematical model of inhibitory growth control

We construct a model that assumes an inhibitory NGF-dependent signal within the ganglion, integrated with known signalling components of NGF growth promotion, and that satisfies the constraints of the experimental data. Our approach is motivated by the seminal signalling models of ref. [28], who demonstrated that a network architecture that combines competing activating and inhibitory pathways with upstream signal amplification is sufficient to explain perfect adaptation and high sensitivity in amoebae and neutrophils. We find that, in a different region of parameter space, a similar network structure also embodies the minimal ingredients required to explain growth and gradient sensing in our system.

We begin by treating a neurite as a single well-mixed compartment that receives two NGF-dependent inputs. One input is assumed to be transduced by receptors at the growth cone, and the other by the proposed inhibitory signal at the cell body. Although in reality this system is more complex, involving, for instance, transport along an extending neurite, our first objective was to determine sufficient processes by which the two primary inputs can be integrated to explain the data. With these simplifications, we initially construct a model that accounts for the biphasic NGF dependence of ganglion outgrowth, but is unable to simultaneously satisfy the requirements of gradient detection. We then construct a signalling network for gradient detection that sensitively compares two concentrations, independent of their magnitude and associated signal saturation. Finally, we couple these two modules together in a two-compartment model, in which we explicitly include transport of signalling components between growth cone and cell body. Simulating this network in neurites extending in a gradient, we account for all competing demands of the experimental data.

### Model 1: Growth

**Fig 3:**
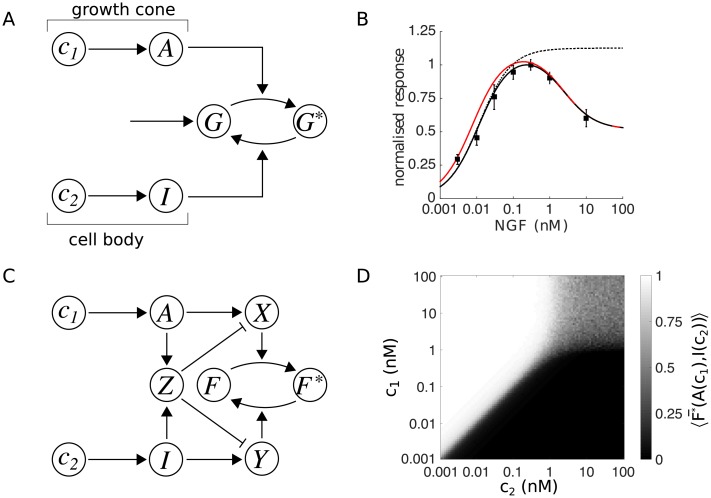
Signalling networks for neurite growth and gradient sensing. Activating (*A*) and inhibitory (*I*) signalling pathways are stimulated in parallel by NGF at the growth cone *c*_1_, and within the ganglion *c*_2_. By sensing an asymmetry in concentrations between growth cone and cell body, an extracellular gradient can be detected. (A) Model 1: growth. The signals are integrated by conversion of a substrate *G* to a growth promoting active form G*. (B) The steady-state output of the growth model, ($\bar{G^{*}}$, black line, normalised by maximum value) reproduces the biphasic ganglion outgrowth response in uniform concentrations (*c*_1_ = *c*_2_), and saturating dissociated cell response (*c*_2_ = 0, dotted line). Black squares are data replotted from Fig 1C, normalised by the maximum outgrowth at 0.3 nM. Due to these two constraints, the network lacks the sensitivity for gradient detection. The model response with a 50% asymmetry in inputs (*c*_1_ = 1.5*c*_2_, *c*_2_ defined by the x-axis; red line) shows only a small increase over the uniform condition. Parameters: *k*_0_ = 1, *k*_1_ = 100, *k*_2_ = 0.75 and *K*_*I*_ = 5 nM. (C) Model 2: gradient detection. Signal integration occurs through intermediaries *X* and *Y* that convert a protein *F* to an active form F*. Dual negative regulation from an interaction between *A* and *I* yields a sensitive readout of the ratio *c*_1_/*c*_2_ in the steady-state output. (D) Gradient detection is robust to parameter perturbations for concentrations of 0.001 − 1 nM. For each pair (*c*_1_, *c*_2_), the average response over 100 trials with noisy parameters is shown. An output of $\bar{F^{*}}=0.5$ indicates the ‘decision’ that *c*_1_ = *c*_2_, $\bar{F^{*}}>0.5$ indicates *c*_1_ > *c*_2_, and $\bar{F^{*}}<0.5$ indicates *c*_1_ < *c*_2_. Parameters as described in *Methods*.

We consider activating (*A*) and inhibitory (*I*) signals that interact within a cell to regulate the conversion of a substrate *G* to a growth promoting active form G* (Fig 3A). We construct the activating pathway as a coarse-grained representation of known NGF/TrkA receptor signalling. The signal *A* represents the binding occupancy of TrkA receptors at the growth cone, which drives growth in response to the local NGF concentration *c*_1_. We assume saturable binding, such that the steady-state of *A* is given by the standard expression

$$\begin{array}{c} \bar{A}\left(c_{1}\right)=A_{T}\frac{c_{1}}{c_{1}+K_{A}}, \end{array}$$ (1)

with *A*_*T*_ the total number of receptors on the growth cone and *K*_*A*_ the dissociation constant. Consistent with measured values of ∼ 0.01 − 1 nM [29–31], we fix *K*_*A*_ = 0.1 nM, and use an order of magnitude estimate of *A*_*T*_ = 1000 total receptors. For the proposed inhibitory pathway, we do not model possible processes of secretion or cell-cell communication explicitly. Working under the assumption that these are short-range effects between neighbouring cells, we simply model the signal *I* as responding to the local NGF concentration *c*_2_ at the cell body within the ganglion. As, at some stage, this must be transduced by receptor binding, we coarse-grain this signal into a similar form to that of *A*,

$$\begin{array}{c} \bar{I}\left(c_{2}\right)=I_{T}\frac{c_{2}}{c_{2}+K_{I}}. \end{array}$$ (2)

We assume for simplicity that *I* has the same maximal intensity as *A*, achieved straightforwardly in the model by setting *I*_*T*_ = *A*_*T*_, and leave *K*_*I*_ as a free parameter. We return to discuss the interpretation of this signal in *Discussion*.

Integration of the activating and inhibitory signals is modelled as a simple push-pull reaction. The substrate is produced in the inactivated form at a constant rate, activated in proportion to *A* and inactivated in proportion to *I*, and decays exponentially in either form. The output of the model is the concentration of protein in the active form G*, which we assume acts linearly to control neurite extension. We provide the governing differential equations and parameters of the model in *Methods*.

We compute the steady state of the network under the assumption that the input concentrations remain fixed (ignoring for now the change in growth-cone concentration during neurite extension in a gradient). Expressed in terms of the signals of Eqs (1) and (2), the steady-state output is given by

$$\begin{array}{c} \bar{G^{*}}=\frac{k_{0}\bar{A}}{k_{1}+k_{2}\bar{A}+\bar{I}}, \end{array}$$ (3)

where the constants *k*_0_, *k*_1_ and *k*_2_ are combinations of rate parameters. When *c*_1_ = *c*_2_, fitting of parameters *k*_*i*_, along with *K*_*I*_ from Eq (2), yields a response in good agreement with the NGF dependence of explant outgrowth (Fig 3B). Setting *c*_2_ = 0, to remove the influence of inhibitory signalling, gives a simple model for dissociated cell growth that exhibits the saturating response of Fig 1F. Thus, the mechanism we propose, expressed as a very simple model, quantitatively accounts for the results of our experiments.

By itself, however, this model lacks the gradient sensitivity implied by the experiments of ref. [18]. We illustrate this in Fig 3B, in which we plot the response of the constrained model with a 50% asymmetry in inputs (*c*_1_ = 1.5*c*_2_; red line). Although this is nearly double the maximum value experienced by neurites in the experiments of ref. [18], only a modest increase in response compared to the uniform condition is observed.

### Model 2: Gradient detection

The obstruction to gradient sensitivity in Model 1 is the saturable form of the signals *A* and *I*, combined with the parameter requirements of a biphasic growth response. Within the framework of the model, sensitive gradient sensing requires a comparison of *A* and *I* while both are in the linear regime with respect to concentration. In this case, *A*/*I* ≈ *c*_1_/*c*_2_, and a highly effective gradient detector can be constructed. Indeed, a central assumption of the models of ref. [28] is that both activating and inhibitory signals are far from saturation. Here, however, a biphasic steady-state response (Fig 3B) requires that activation occurs at much lower concentrations than inhibition. This imposes the necessary condition that *K*_*A*_ ≪ *K*_*I*_, precluding a direct comparison in respective linear regimes when the difference in input concentrations is small. Independent of the model, TrkA activation by NGF is indeed saturable [29–31], yet remarkable gradient sensitivity was observed experimentally over two orders of magnitude of concentration (Fig 2C). Thus, both the model and experimental data point to a mechanism for gradient detection that operates with high sensitivity, despite receptor saturation.

How can the effects of signal saturation be overcome? A common theoretical assumption is that cells can perform the necessary computations to invert expressions such as (1) and (2), and thus access the original input variables [32–36]. In this way, a system that depends on the ratio of inputs could be constructed by forming the expression

$$\begin{array}{c} \frac{K_{A}\bar{A}\left(I_{T}-\bar{I}\right)}{K_{I}\bar{I}\left(A_{T}-\bar{A}\right)}=\frac{c_{1}}{c_{2}}, \end{array}$$ (4)

providing a possible means for sensitive gradient detection. However, to the best of our knowledge, no biologically realisable implementation of this operation has been derived. To make our hypothesis concrete, we construct a network that performs the algebraic manipulations required of Eq (4) (motivated by ref. [37]), and thus present an explicit gradient sensing mechanism. To do so requires only minor modifications of Model 1, sharing both the input pathways and basic network structure (Fig 3C). A dual negative regulation, induced by interaction between *A* and *I*, provides the necessary processing to unpack both saturating signals simultaneously.

In this network, the activating and inhibitory pathways are integrated indirectly through downstream effector molecules *X* and *Y*. The two effectors enzymatically convert a target protein between an inactive *F* and active form F*. Inhibitors *Z*_*X*_ and *Z*_*Y*_ are produced through upstream interaction between *A* and *I*, and thus degrade the effectors in proportion to the product *AI*. Intuitively, this can be understood as a form of mutual inhibition where *A* acts to suppress the activity of *I*, when *I* is present, and vice versa. We provide the governing differential equations and parameters of the model in *Methods*. At steady-state, and assuming that degradation of *Z*_*X*_ and *Z*_*Y*_ is much slower than the inhibitory reactions,

$$\begin{array}{c} \bar{X}\approx k_{3}\bar{A}\left(k_{4}-\bar{I}\right)\;\text{and}\;\bar{Y}\approx k_{5}\bar{I}\left(k_{6}-\bar{A}\right), \end{array}$$ (5)

where the *k*_*i*_ are combinations of rate parameters, and equality holds when the rates of inhibitor degradation go to zero. Assuming general Michaelis-Menten kinetics for activation and inactivation of *F*, the steady-state output of the network $\bar{F^{*}}$ is given by the Goldbeter-Koshland function [38], which depends only on $\bar{X}$ and $\bar{Y}$ through the ratio $\bar{X}/\bar{Y}$. Thus, with appropriate choice of parameters *k*_*i*_, by comparison with Eq (4), the network output is a function of the concentration gradient, and independent of background concentration and associated receptor saturation. Moreover, if the enzyme kinetics are assumed to operate in the zero-order regime, the network can be made arbitrarily sensitive to small differences in concentrations, while remaining bounded in the case that *c*_2_ = 0. Functionally, this is approximately equivalent to the response,

$$\begin{array}{c} \bar{F^{*}}\left(A\left(c_{1}\right),I\left(c_{2}\right)\right)∼\frac{{\left(c_{1}/c_{2}\right)}^{h}}{1+{\left(c_{1}/c_{2}\right)}^{h}}, \end{array}$$ (6)

with tunable Hill coefficient *h*.

To perfectly extract the gradient signal through this network requires an appropriate choice of the parameters that appear in Eq (5), such that *k*_4_ = *I*_*T*_, *k*_6_ = *A*_*T*_ and *k*_3_/*k*_5_ = *K*_*A*_/*K*_*I*_. To test the robustness of the output to changes in these values we computed the steady-state with random perturbations to parameters. For individual pairs of inputs (*c*_1_, *c*_2_) spanning 0.001 − 100 nM, a 10% multiplicative, uniformly distributed noise term η∼U(0.9,1.1) was applied independently to each parameter in Eq (5). We performed this procedure 100 times for each pair of concentrations, and computed the average output over trials $\langle \bar{F^{*}}\left(A\left(c_{1}\right),I\left(c_{2}\right)\right)\rangle$ (Fig 3D), representing the average response of a collection of neurites with some cell-cell variability in intrinsic parameters. For concentrations between 0.001 − 1 nM, a sharp separation persists between up-gradient (*c*_1_ > *c*_2_) and down-gradient (*c*_1_ < *c*_2_) conditions, with an eventual loss of discriminability at higher concentrations. Further simulations revealed *k*_6_ to be the most sensitive parameter, as keeping this value fixed extended the sharp boundary to concentrations up to 100 nM.

### Coupled growth and gradient detection

We have shown how an inhibitory cell-body signal can be integrated with TrkA activation at the growth cone to produce two distinct outcomes. The push-pull network of Model 1 reproduces the biphasic ganglion outgrowth response, whereas the dual negative regulation of Model 2 yields an adaptive sensor that is highly sensitive to small differences in input concentrations. We now couple these motifs together to produce a model of NGF signalling that quantitatively accounts for the experiments of refs. [18, 22].

**Fig 4:**
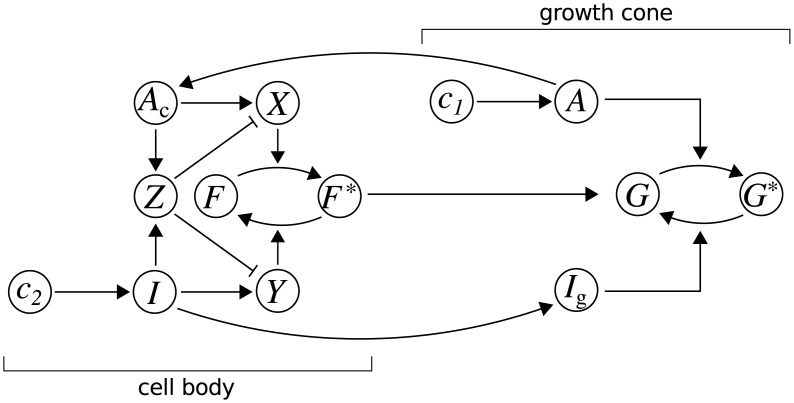
Coupled growth and gradient detection. The motifs depicted in Fig 3A and 3C are coupled together in a two-compartment model of a neurite. Activated receptors *A* are retrogradely transported from the growth cone to cell body to form a population *A*_c_, and the inhibitory signal *I* is anterogradely transported to the growth cone to form a copy *I*_g_. The output of the cell-body compartment F* regulates the supply of the substrate *G* at the growth cone.

For the coupled system, we model a neurite as two well-mixed compartments, representing the growth cone and cell body. The network of Model 1 is localised to the growth cone, whereas the network of Model 2 is localised to the cell body (Fig 4). Communication between compartments occurs via retrograde transport of activated receptors *A* to form a cell body population *A*_c_, and anterograde transport of the inhibitory signal *I* to produce a copy at the growth cone *I*_g_. Similar to the signal amplification by substrate supply of ref. [28], when the output of the cell-body compartment F* regulates the synthesis and transport of growth cone substrate *G*, neurites extend preferentially in the direction of a gradient.

**Fig 5:**
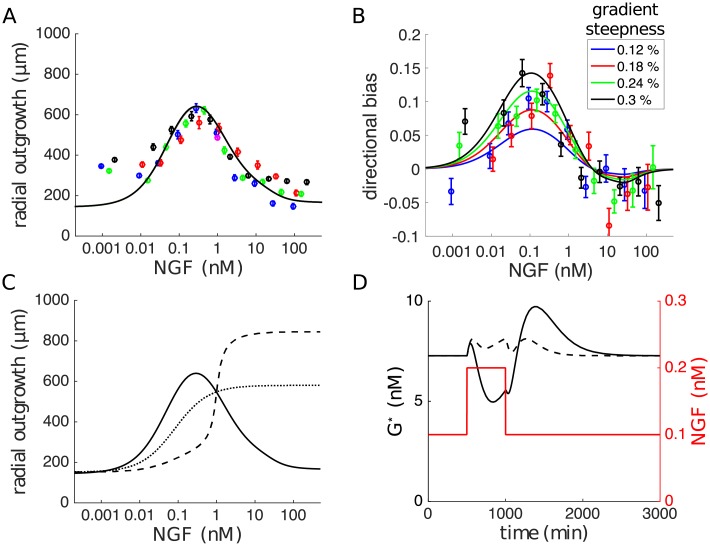
Model simulations and predictions. (A,B) Simulations of the model yield good agreement with experimental data, reproducing both the biphasic average radial outgrowth (A) and sensitive gradient response (B). Coloured markers are data replotted from Fig 2, error bars are SEM. (C) Predicted relief of growth inhibition when distal neurites are exposed to high NGF concentrations while the ganglion body is held at 1 nM. The full model exhibits a switch-like response as the distal neurite concentration exceeds that of the ganglion body (dashed line). Without the gradient sensor, the model response is less steep (dotted line). The biphasic curve from (A) is replotted for comparison (solid line). (D) Predicted temporal response to a 500 min pulse of NGF. A transient global doubling of NGF concentration (red line) produces a large out of phase change in growth rate (solid black line) due to time delays from transport. Increasing the rates of transport suppresses this effect (dashed black line). Parameters used in the simulations are given in Table 1.

We tested the model against the gradient data set by simulating 48 h ganglion outgrowth in shallow exponential gradients (Methods). Fitting the parameters of the model (Table 1) yielded good agreement with the data over all concentration and gradient conditions tested. The average radial outgrowth of simulated explants follows the characteristic biphasic NGF dependence, and is independent of the gradient steepness (Fig 5A). The directional bias of simulated outgrowth also closely matches that of the experimental data, exhibiting a large asymmetry in outgrowth for background concentrations of 0.01 − 1 nM (Fig 5B). Thus, for the first time, we have provided a mechanistic and quantitative explanation of these two fundamental features of neurite growth control. Modular processing, coupled by protein transport and supply, permits powerful integration of antagonistic signals and fine tuning of collective growth.

**Table 1:** Model parameters for coupled growth and gradient detection.

Parameter	Value	Parameter	Value
*A*_*T*_	1000	*I*_*T*_	1000
$k_{+}^{A}$	1 nM^−1^ min^−1^	$k_{-}^{A}$	0.1 min^−1^
$k_{+}^{I}$	0.01 nM^−1^ min^−1^	$k_{-}^{I}$	0.1 min^−1^
*k*_r_	0.0025 min^−1^	$k_{\text{deg}}^{A}$	0.005 min^−1^
*k*_a_	0.0025 min^−1^	$k_{\text{deg}}^{I}$	0.005 min^−1^
*α*_0_	0.1 nM min^−1^	*β*_0_	0.01 min^−1^
*α*_1_	5 × 10^−6^ nM^−1^min^−1^	*β*_1_	2.5 × 10^−4^min^−1^
*α*_2_	0.01 nM min^−1^	*β*_2_	1 min^−1^
*α*_3_	0.01 nM min^−1^	*β*_3_	$\frac{\alpha_{5}b_{2}K_{A}}{\alpha_{4}K_{I}}$ min^−1^
*α*_4_	$\frac{\alpha_{2}}{I_{T}}$ nM min^−1^	*β*_4_	1 × 10^−5^min^−1^
*α*_5_	$\frac{\alpha_{3}k_{\text{deg}}^{A}}{A_{T}k_{\text{r}}}$ nM min^−1^	*β*_5_	1 × 10^−5^min^−1^
*α*_6_	1 min^−1^	*β*_6_	1 min^−1^
*γ*_1_	0.01 nM^−1^ min^−1^	*γ*_2_	0.01 nM^−1^ min^−1^
*α*_*F*_	0.06 min^−1^	*F*_*T*_	10 nM
*K*_*X*_	1 nM	*K*_*Y*_	1 nM
*r*_0_	0.05 μm min^−1^	*k*_ext_	0.02 μm nM^−1^ min^−1^

### Model predictions

There are two key structural features of the model that contribute to the NGF response. The first is our central hypothesis that NGF-dependent inhibition arises from signalling within the ganglion body. The second is that detection of shallow gradients occurs via transport and comparison of signals between the growth cone and cell body, thus maximising the concentration differences being sensed. We describe two experiments which could test these claims, and simulate the model to predict the observable response.

The role of inhibitory signalling within the ganglion can be tested by growing explants in compartmentalised chambers that separate neurite and cell-body regions, similar to the assay of ref. [39]. With a fixed NGF concentration at the cell bodies, the model predicts an absence of growth inhibition when high concentrations are applied to the distal neurites (Fig 5C). Moreover, due to the sensitivity of gradient detection, the model predicts a switch-like transition to this regime. In the absence of gradient sensing (setting the output of this component of the model F* to a constant), the transition is less steep, but the predicted outgrowth remains uninhibited as the distal neurite concentration is increased (Fig 5C).

As NGF/TrkA retrograde transport is slow compared with receptor binding and the rate of neurite growth [40], the necessity of this mechanism for gradient detection can be tested with temporal manipulations. Fig 5D shows a simulation of the model in a uniform 0.1 nM concentration which was transiently increased to 0.2 nM with a 500 min pulse of NGF. Because there is a time delay in transporting newly bound receptors from the growth cone, the concentration at the cell-body is initially perceived as higher, creating an artificial negative gradient. The model therefore predicts, counterintuitively, that a uniform concentration increase will transiently decrease the rate of neurite outgrowth (Fig 5D). Similarly, a concentration decrease is predicted to have the opposite effect. The predicted out of phase response is robust to the precise temporal regulation of NGF, requiring mainly that the timescale is equivalent to that of retrograde transport, or slower (≈400min or more).

## Discussion

Wiring the nervous system requires the coordinated transduction of a diverse array of chemical signals. We have shown how integration of antagonistic pathways can exert fine control of neurite growth and guidance. We first sought to clarify the contested origin of the biphasic trophic effects of NGF, and found previous hypotheses were unsupported by our experiments with rat DRG explants and dissociated cells. Our results show that growth inhibition is a collective property of the intact ganglion, however, this is not a consequence of increased fasciculation of extending neurites. We thus proposed a role for inhibitory signalling within the ganglion body, and demonstrated with a mathematical model that this mechanism can account for both trophic inhibition in high concentrations, and extreme tropic sensitivity in very shallow gradients. Constraining the response of the model with extensive experimental data, we derived testable predictions with which the role of inhibitory signalling can now be rigorously explored.

### Trophic inhibition

What is the molecular basis of the mechanism we describe? As growth inhibition was observed only for explants, but not for dissociated cells, our experiments provide strong evidence against any pathway in which inhibitory effects are mediated by direct binding of NGF to cell-surface receptors. This includes the proposal of ref. [16] that excess NGF stabilises the population of TrkA receptors in an inactive configuration, as well as the suggestion that TrkA receptors undergo activity-dependent down regulation analogous to chemotactic receptors in leukocytes [21]. Similarly, the lack of effect in dissociated cells precludes other possible hypotheses such as direct inhibition by the low-affinity NGF receptor p75, or toxicity from overstimulation of downstream TrkA pathways. This led us to propose the involvement of paracrine signalling within the ganglion by NGF-dependent secretion and subsequent binding of an inhibitory factor.

In a period of competitive survival in sympathetic neurons, NGF signalling leads to cell-body secretion of brain derived neurotrophic factor, which promotes apoptosis of neighbouring cells through the receptor p75 [25]. Although p75 is also an antagonist of NGF/TrkA growth signalling, whether it plays a general role in inhibition in high concentrations is unclear; genetic knockout of p75 had no observable effect on DRG explants in bath applications of NGF [19], whereas outgrowth from trigeminal ganglia was no longer repelled from NGF-coated beads [20]. However, p75 belongs to the broader tumour necrosis factor receptor superfamily, of which many members influence neurite growth in critical developmental stages [41–47]. Shared signalling pathways among this family of receptors suggest a potential general basis for paracrine growth regulation.

In DRGs, tumour necrosis factor receptor-1 (TNFR1) is localised to neuronal cell bodies, and strongly antagonises NGF/TrkA responses when stimulated by tumour necrosis factor alpha (TNF_*α*_) [48]. A source of secreted TNF_*α*_ in DRGs is the satellite glial cells that closely ensheathe neuronal cell bodies, and express both p75 and TrkA receptors [26]. Although the role of NGF receptors in satellite glial cells is only beginning to be understood [26, 49], it is plausible that high NGF concentrations could produce the glial cell activation that elicits TNF_*α*_ release, and thus a cell-body inhibitory signal through TNFR1. Consistent with this prediction, genetic knockout of either TNF_*α*_ or TNFR1 yielded a threefold increase in embryonic DRG explant outgrowth at an NGF concentration of ≈ 2 nM [48].

Integration of NGF and TNF_*α*_ signalling could occur via the Akt pathway, analogous to DRG neurons stimulated with related neurotrophin insulin-like growth factor (IGF) [50]. High concentrations of TNF_*α*_ antagonise Akt activation, growth associated protein 43 (GAP43) expression and neurite growth promoted by IGF, in a phosphatidylinositol 3-kinase (PI3K)-dependent manner [50]. PI3K/Akt signalling is a primary pathway of retrograde TrkA activity [51, 52], and GAP43 activation by TrkA at the growth cone promotes cytoskeletal assembly and growth [53], suggesting possible candidates for the substrates *F* and *G* in the model. Thus, an interpretation of the signalling network of Fig 4 is that TrkA and TNFR1 control activation of Akt at the cell body (*F* ↔ F*), regulating expression of GAP43, which is then activated at the growth cone (→ *G* → G*). A second pathway of TNFR1 signalling involves a cascade of several caspases. Caspase- 3,6 and 9, in particular, are key effectors of the axonal degeneration that accompanies NGF withdrawal [54, 55]. GAP43 is a substrate of caspase-3 [56], thus providing a link between TNFR1 activation at the cell body, and growth inhibition at the growth cone (*G* ← G*). These molecular candidates provide further means by which the proposed role of TNF_*α*_/TNFR1 signalling can be tested experimentally.

### Tropic sensitivity

The sensitivity of explant outgrowth to shallow gradients observed experimentally by refs. [17, 18, 22, 57, 58] demonstrates the exquisite chemosensory ability of developing neurites. In our model, this is explained by a comparison of concentrations between growth cone and cell body, and a chemical computation that overcomes the deleterious effects of receptor saturation.

Our approach was inspired by the study of ref. [28]. There it was shown how the chemosensory system of a single cell can exhibit a steady-state response to uniform extracellular concentration changes that is independent of the concentration (perfect adaptation), yet maintain a persistent asymmetry of response across the cell when presented with a gradient. Perfect adaptation arises through competing processes of activation and inhibition, similar to Fig 3A in the case that *c*_1_ = *c*_2_, and non-saturating kinetics that permit a cancellation of concentration-dependent components in the steady-state output. Given the adaptation constraint, sensitive gradient detection was achieved in ref. [28] through a global signal conveyed by diffusion of the inhibitor, and feedback that increases the supply of protein. The behaviour and constraints of our model are rather different; ours is an extended multicellular system, exhibits a biphasic dose response rather than perfect adaptation, and requires that signals are transduced sensitively by receptors that are known to saturate within the regime of interest. Yet, consistent with the prediction of ref. [28] that the principles underlying their work would be conserved across systems, we found that a similar network design and substrate supply mechanism also provided a compatible model structure.

The presence of receptor saturation in our model is the key difference that introduces concentration dependence into the steady-state response, which has the appropriate biphasic form when *K*_*I*_ ≫ *K*_*A*_. However, this constraint also demands a distinct treatment of gradient detection as it limits the effectiveness of directly comparing activator and inhibitor. By introducing a dual negative regulation of opposing enzymes to counter saturation, we showed how tropic sensitivity can, in principle, persist over a wide range of concentrations. Although serving a different purpose in our model, we note that this additional interaction also permits perfect adaptation, and thus provides a simple generalisation of the work of ref. [28] to encompass the wider concentration regime in which signals saturate. Whether this interaction is indeed a feature of NGF signalling is unknown. Intriguingly, PI3K and the opposing enzyme phosphatase and tensin homology (PTEN), are subject to a dual positive regulation by regulatory subunit p85 [59]. This suggests a possible implementation of a variant of the motif by sequestration of p85, though to pursue this idea is beyond the scope of the present work. To allow comparison of concentrations from different points in space, our model assumes trafficking of components along the neurite, rather than the intracellular diffusion of inhibitor assumed by ref. [28]. Importantly, this also allows the growth and guidance components of the model, which have competing computational demands, to be processed in distinct regions of the cell. Here, the substrate supply mechanism does not amplify these signals through feedback, but couples them to provide the fine modulation required of guidance by differential growth.

Although the spatial structure of a neuron largely precludes intracellular diffusion as a means of relaying signals in our system, another possibility is that this could occur extracellularly. The cell-cell interactions within our model are assumed to be of short range, with an inhibitory signal that depends only on the NGF concentration local to the cell body (Eq (2)). However, a multicellular counterpart of the global inhibition mechanism of ref. [28] was recently proposed in which an inhibitory factor diffuses throughout a collection of cells [60, 61]. In this, collective signalling permits a robust response to a gradient, even at sensory limits at which the stimulus cannot be detected by an isolated cell. Thus, although global signalling is not required for gradient detection within our model, long-range diffusion within a ganglion could serve as an alternate or additional means of amplifying a weak gradient across the collection of cells. For long diffusion length scales, such a mechanism would also likely yield a dependence of trophic response on explant body size, as the volume from which the hypothetical inhibitor would be secreted grows more quickly with the radius than the boundary over which the inhibitor would escape by diffusion. However, we observed no correlation between outgrowth and explant body size in the experimental data (S2 Table), arguing against this possibility. To examine these effects more precisely requires detailed modelling of diffusion within the crowded environment of a ganglion, suggesting another avenue for future work, should our key predictions (Fig 5C and 5D) be confirmed.

The guidance mechanism we propose is of a very different nature to that originally posited for shallow gradients by refs. [18, 22]. These studies consider a Bayesian model in which guidance decisions are drawn from a probability distribution, determined by computation of gradient direction from noisy receptor binding. Here, we do not consider binding noise; given the 48 h duration of the shallow gradient experiments, quantified by measurements that average over many neurites, we believe our deterministic model gives a suitable account of collective outgrowth, into which noise can be absorbed. Although our model supports the suggestion of ref. [22] that shallow gradients are sensed along the neurite length, our approach leads to a different conclusion regarding the decline in tropic response magnitude with high background concentrations. The interpretation of refs. [18, 22] was that this is due to receptor saturation limiting the signal to noise ratio for reliable detection. However, our model yields a similar response, while explicitly removing the effect of receptor saturation. Instead, we suggest that in background concentrations with low average outgrowth, neurites simply do not extend far enough through the gradient to develop a concentration asymmetry along their length sufficient to drive a large tropic response. The biphasic average outgrowth constraint was not enforced in simulations that employed the Bayesian model [22, 58], meaning this additional dependence may not have been observed.

What is the developmental benefit of this mode of tropic response? Navigation over long distances towards a chemoattractant source may require an early phase of growth in gradients too shallow for a reliable turning response. One possibility for overcoming this problem is that growth cones synergistically process gradients of multiple cues [62]. Another is that a comparison of growth cone and cell-body concentrations may initially orient collective extension toward the source, and thus avoid expensive outgrowth in the wrong direction. As the gradient becomes steeper, closer to the source, turning responses could then take over to direct individual neurites at finer spatial scales. Even at these later stages, a cell-body signal could serve as a useful reference concentration, acting to halt the growth of neurites that have strayed too far off course.

### Summary

Our aim in this study was to understand better the origins and implications of NGF regulation of neurite growth and guidance. We provided experimental evidence that demands a revision of current theories of growth, and argued that theories of guidance in shallow gradients should address the coupling to the underlying growth response. The specific hypothesis we proposed is centred around a role for inhibitory signalling at the neural cell bodies, and quantitatively explains multiple features of the observed responses of developing neurites. The predictions of our theory, which can be tested both with macroscopic manipulations of growth conditions and targeting of specific candidate molecules, motivate new experiments to unravel the complex processes of nervous system development and repair.

## Methods

### Ethics statement

Experiments involving animals were conducted in accordance with the Animal Care and Protection Act Qld (2002), and the Australian Code of Practice for the Care and Use of Animals for Scientific Purposes, 8th edition (2013). Ethics approval was obtained from the University of Queensland Anatomical Biosciences Ethics Committee, approval QBI/548/16.

### Neurite growth assay

Thoracic and lumbar DRGs were extracted from P1-P2 rat pups into Leibovitz medium on ice. Excess axonal tissue was trimmed off. For the explants, DRGs were digested for 12 min in 0.25% trypsin at 37°C and then washed three times in Leibovitz and kept at 4°C until use. For the dissociated cells, DRGs were digested for 45 min in 0.25% trypsin at 37°C and then triturated through a fire-polished pipette. Cells were washed three times in opti-MEM and concentrated by centrifugation to a smaller volume (≈ 200 μL).

Collagen was prepared, on ice, with the following concentrations: 0.2% rat tail collagen type 1 (Corning), 0.1% sodium bicarbonate, 1×opti-MEM and 1×penicillin/streptomycin. After addition of NGF to the collagen, the dissociated cells were added to the collagen and mixed thoroughly. 750 μL of the collagen was spread on a 35 mm petri dish and allowed to set. A second layer of 750 μL of collagen was added and 5 − 12 DRG explants were added within this second layer. After the collagen set, the dishes were transferred to an incubator for 2 days (37°C and 5% CO_2_).

### Immunostaining and microscopy

Explants and dissociated cells embedded in collagen were fixed with 4% paraformaldehyde/0.1% Triton-X 100 in PBS overnight. Plates were washed five times with PBS with 1 hour between washes, and then incubated overnight at 4°C with *β*-III-tubulin antibody TuJ1 (1:500; R&D Systems). After five washes with PBS of 1 hour each, plates were incubated overnight at 4°C with secondary antibody Alexa Flour 488-conjugated goat anti-mouse IgG (1:500, Invitrogen). Plates were washed five times in PBS for 1 hour each before acquisition of images using Apotome imaging on a Zeiss Z1 microscope. Images were acquired as z-stacks and flattened by average intensity projection for explants, and by maximum intensity projection for dissociated cells. For measurements of fasciculation, additional z-stacks were acquired at higher resolution (0.645 μm per pixel), and flattened by average intensity projection.

### Quantification of neurite growth

Explant images were manually segmented (using ImageJ) to separate the cell-body and neurite outgrowth regions, and thresholded by pixel intensity to form binary masks, as described in ref. [18]. For each image, boundary curves were fitted to the cell-body mask and the outer boundary of the largest connected component of the outgrowth mask using the MATLAB function ‘bwboundaries’. The curves were smoothed with a moving average filter of width 150 pixels, and then parameterised by polar coordinates with *N* = 360 discrete angles $\theta_{n}=\frac{2\pi n}{N}$ about an origin defined as the centroid of the cell-body mask. In the event that a ray from the origin intersected a boundary at multiple points, the closest point to the origin was selected. The radial outgrowth function, *R*(*θ*_*n*_), was defined as the distance between the cell-body and neurite region boundaries at each *θ*_*n*_. We extended this to a continuous representation by performing a discrete Fourier transform to give *R*(*θ*_*n*_) in terms of frequency components, $R\left(\theta_{n}\right)=\Sigma_{k=0}^{N-1}{\hat{R}}_{k}\cdot e^{2\pi ikn/N}$, and then folding about the Nyquist frequency to determine equivalent Fourier coefficients as $a_{0}=\text{Re}\left({\hat{R}}_{0}\right)$, $a_{k}=2\text{Re}\left({\hat{R}}_{k}\right)$ and $b_{k}=-2\text{Im}\left({\hat{R}}_{k}\right)$, 1 ≤ *k* ≤ 180. Examples of the image processing steps are shown in S3 Fig.

We found that the first five coefficients were sufficient to reconstruct the major features of explant outgrowth via

$$\begin{array}{c} R\left(\theta \right)\approx a_{0}+a_{1}\text{cos}\left(\theta \right)+b_{1}\text{sin}\left(\theta \right)+a_{2}\text{cos}\left(2\theta \right)+b_{2}\text{sin}\left(2\theta \right). \end{array}$$ (7)

The coefficient *a*_0_ determines the average radial outgrowth, *a*_1_ and *b*_1_ determine the bias in outgrowth in orthogonal image axes, and *a*_2_ and *b*_2_ capture the polarised growth exhibited by some explants (likely resulting from growth hotspots at the sites of axotomy). For data analysis, we used the coefficient *a*_0_ to quantify average outgrowth (in units of μm). For the NGF gradient data set, we used the normalised coefficient *b*_1_/*a*_0_ as a dimensionless measure of outgrowth bias up the gradient.

Neurite growth from dissociated cells was quantified by manual tracing using the ImageJ plugin NeuronJ [63]. We recorded the length of the longest neurite of each cell as an analogue of the extent of radial outgrowth recorded for the explants.

### Quantification of fasciculation

Neurite bundle widths were measured using the ImageJ plugin Ridge Detection [64, 65]. For each explant, we applied the analysis to four 650 μm × 100 μm image strips. The strips were arranged at distances of 150 μm from the explant body, with the short axes parallel to the predominant direction of neurite extension (approximately forming a square about the body). Detected neurite segments of length less than 20 μm were excluded. These restrictions limited double counting of neurites that were identified by Ridge Detection as a collection of broken segments, and artefacts from the regions of dense growth near the explant body. Examples of the segmentation are shown in S2A Fig. Pooling across explants in each condition (0.3 nM and 10 nM NGF) we constructed distributions of neurite bundle widths for statistical comparison. As a positive control, we constructed sample image sets of patches containing mostly thin or thick bundles (S2B and S2C Fig), which were easily discriminated by the image analysis. We used the width parameter *σ* = 1.37 pixels in the Ridge Detection algorithm (which sets an effective range for detection and width estimation), and confirmed that changing this parameter did not effect the difference in distributions of tested conditions, nor positive controls.

### Model equations

#### Activation and inhibition

We consider activating and inhibitory pathways that are stimulated by the concentration of NGF at the growth cone *c*_1_, or cell body *c*_2_, respectively. The activating signal *A* represents the occupancy of TrkA receptors at the growth cone, for which we assume first-order binding kinetics. We use an analogous equation for the proposed inhibitory signal *I*. We model these two processes with the pair of ordinary differential equations

$$\frac{dA}{dt}=c_{1}k_{+}^{A}\left(A_{T}-A\right)-k_{-}^{A}A$$ (8)

$$\frac{dI}{dt}=c_{2}k_{+}^{I}\left(I_{T}-I\right)-k_{-}^{I}I.$$ (9)

In Eq (8), $k_{+}^{A}$ and $k_{-}^{A}$ are the respective rate constants for binding and unbinding of NGF molecules, and *A*_*T*_ denotes the total number of receptors on the growth cone. We use the measured value for the unbinding rate $k_{-}^{A}=0.1{\text{min}}^{-1}$ [66], and set $k_{+}^{A}=1\;\text{n}{\text{M}}^{-1}\;{\text{min}}^{-1}$, such that the dissociation constant $K_{A}=k_{-}^{A}/k_{+}^{A}=0.1\;\text{nM}$ as described in *Results*. Eq (9) is a phenomenological representation, used for concreteness, that yields a similar steady-state form to that of *A*. The precise dynamics have only a minor influence on our results, as the cell-body concentration *c*_2_ remains fixed in the majority of our simulations.

In Model 1 and Model 2 (Fig 3A and 3C), which we use to determine appropriate network structures, we treat a neurite as a single compartment that receives the two inputs described by Eqs (8) and (9). When extending to the coupled model (Fig 4), we also explicitly include retrograde transport of activated growth-cone receptors to form a cell body population *A*_c_, and anterograde transport of the signal *I* to form a copy at the growth-cone *I*_g_. Transport is modelled with the Eqs

$$\frac{dA_{\text{c}}}{dt}=k_{\text{r}}A-k_{\text{deg}}^{A}A_{\text{c}}$$ (10)

$$\frac{dI_{\text{g}}}{dt}=k_{\text{a}}I-k_{\text{deg}}^{I}I_{\text{g}}.$$ (11)

In Eq (10), *k*_r_ is the rate of retrograde transport, and *k*_deg_ is the rate of degradation of the cell-body population of receptors. Trafficking of receptors along the neurite occurs via the fast transport system, at a rate of 2 − 5 μm sec^−1^ [40]. The rate limiting step of retrograde TrkA transport is instead their internalisation and packaging into endosomes [40], yielding a steady-state flux from growth cone to cell body of 10 − 15% of activated receptors per hour [40]. We therefore neglect the trafficking component, and associated dependence on neurite length, and set *k*_r_ = 0.0025 min^−1^. As the rate of transport is considerably slower than the binding kinetics, we also neglect the depletion of receptors at the growth cone, assuming a background replenishment that maintains a constant total *A*_*T*_. For receptor degradation, we set $k_{\text{deg}}^{A}=0.005\;{\text{min}}^{-1}$, which approximates the measured half-life of 3 h [40]. Eq (11) represents the anterograde transport of *I* or its downstream products to the growth cone, at rate *k*_a_, to form a population *I*_g_ which decays at rate $k_{\text{deg}}^{I}$. As we have no *a priori* constraints with which to choose parameters for the inhibitory signal, we simply set $k_{-}^{I}=k_{-}^{A}$, *k*_a_ = *k*_r_ and $k_{\text{deg}}^{I}=k_{\text{deg}}^{A}$, and leave $k_{+}^{I}$ for model fitting via the dissociation constant *K*_*I*_. In this way we ensure that *I* is operating in the biologically relevant regime, in analogy with *A*, and our predictive power pertains more to signal integration and network topology than finer kinetic details.

#### Model 1: Growth (Fig 3A)

Integration of signals *A* and *I* in the growth model occurs via activation and inactivation of the growth response element *G*. The dynamics are given by the equations

$$\frac{dG}{dt}=\alpha_{0}-\alpha_{1}AG+\beta_{1}IG^{*}-\beta_{0}G$$ (12)

$$\frac{dG^{*}}{dt}=\alpha_{1}AG-\beta_{1}IG^{*}-\beta_{0}G^{*}.$$ (13)

Here, the inactive substrate *G* is produced at a constant rate *α*_0_, and decays exponentially in both the inactive and active forms at rate *β*_0_. The substrate is activated by *A*, and inactivated by *I* with first-order kinetics. We assume that the growth rate of a neurite is a linear function of G*.

We compute the steady-state solution of the model, assuming the input concentrations remain fixed. Setting time derivatives to zero, we obtain the following expression for the steady-state of G*:

$$\bar{G^{*}}=\frac{k_{0}\bar{A}}{k_{1}+k_{2}\bar{A}+\bar{I}},$$ (14)

with *k*_0_ = *α*_0_ *α*_1_/*β*_0_ *β*_1_, *k*_1_ = *β*_0_/*β*_1_, and *k*_2_ = *α*_1_/*β*_1_.

#### Model 2: Gradient detection (Fig 3C)

The model depicted in Fig 3C provides a general mechanism for overcoming signal saturation for sensitive gradient sensing. The network takes two saturating signals *A* and *I*, as given by Eqs (8) and (9), and reports the ratio of input concentrations *c*_1_/*c*_2_ in the steady-state output. In this network, effector molecules *X* and *Y* are produced in proportion to *A* and *I* respectively, and decay exponentially. The two effectors enzymatically convert a target protein between an inactive form *F* and active form F* by Michaelis-Menten kinetics (Eq (19)). Here, *F*_*T*_ denotes the total concentration of protein, and *K*_*X*_ and *K*_*Y*_ are the Michaelis constants for the reactions mediated by *X* and *Y* respectively. Eqs (17) and (18) describe the kinetics of two inhibitors, *Z*_*X*_ and *Z*_*Y*_, induced by an upstream interaction between *A* and *I*. The rates of production are modelled as proportional to the product *AI*, and both decay exponentially. Each inhibitor is assumed to bind irreversibly to its target, thereby sequestering both the effector and inhibitor simultaneously, as described by the final terms in Eqs (15)–(18).

$$\frac{dX}{dt}=\alpha_{2}A-\beta_{2}X-\gamma_{1}Z_{X}X$$ (15)

$$\frac{dY}{dt}=\alpha_{3}I-\beta_{3}Y-\gamma_{2}Z_{Y}Y$$ (16)

$$\frac{dZ_{X}}{dt}=\alpha_{4}AI-\beta_{4}Z_{X}-\gamma_{1}Z_{X}X$$ (17)

$$\frac{dZ_{Y}}{dt}=\alpha_{5}AI-\beta_{5}Z_{Y}-\gamma_{2}Z_{Y}Y$$ (18)

$$\frac{dF^{*}}{dt}=\alpha_{6}\frac{X(F_{T}-F^{*})}{K_{X}+\left(F_{T}-F^{*}\right)}-\beta_{6}\frac{YF^{*}}{K_{Y}+F^{*}}$$ (19)

At steady-state, $\bar{X}$ and $\bar{Y}$ are given by

$$\begin{array}{c} \bar{X}=\frac{1}{2}\left[\frac{\alpha_{4}}{\beta_{2}}\bar{A}\left(\frac{\alpha_{2}}{\alpha_{4}}-\bar{I}\right)-\frac{\beta_{4}}{\gamma_{1}}\right]+\frac{1}{2}\sqrt{{\left[\frac{\alpha_{4}}{\beta_{2}}\bar{A}\left(\frac{\alpha_{2}}{\alpha_{4}}-\bar{I}\right)-\frac{\beta_{4}}{\gamma_{1}}\right]}^{2}+\frac{4\alpha_{2}\beta_{4}}{\beta_{2}\gamma_{1}}\bar{A}} \end{array}$$ (20)

$$\begin{array}{c} \bar{Y}=\frac{1}{2}\left[\frac{\alpha_{5}}{\beta_{3}}\bar{I}\left(\frac{\alpha_{3}}{\alpha_{5}}-\bar{A}\right)-\frac{\beta_{5}}{\gamma_{2}}\right]+\frac{1}{2}\sqrt{{\left[\frac{\alpha_{5}}{\beta_{3}}\bar{I}\left(\frac{\alpha_{3}}{\alpha_{5}}-\bar{A}\right)-\frac{\beta_{5}}{\gamma_{2}}\right]}^{2}+\frac{4\alpha_{3}\beta_{5}}{\beta_{3}\gamma_{2}}\bar{I}}. \end{array}$$ (21)

In the limit that the rate of decay of *Z*_*X*_ and *Z*_*Y*_ is much slower than the rate of the inhibitory reactions (ie. $\frac{\beta_{4}}{\gamma_{1}},\frac{\beta_{5}}{\gamma_{2}}⪡1$), these expressions reduce to

$$\begin{array}{c} \bar{X}\approx \frac{\alpha_{4}}{\beta_{2}}\bar{A}(\frac{\alpha_{2}}{\alpha_{4}}-\bar{I})\;\text{and}\;\bar{Y}\approx \frac{\alpha_{5}}{\beta_{3}}\bar{I}(\frac{\alpha_{3}}{\alpha_{5}}-\bar{A}). \end{array}$$ (22)

With a choice of parameters such that *α*_4_ *β*_3_/*α*_5_ *β*_2_ = *K*_*A*_/*K*_*I*_, *α*_2_/*α*_4_ = *I*_*T*_ and *α*_3_/*α*_5_ = *A*_*T*_, this gives a ratio of *X* and *Y* that approximates the ratio of input concentrations,

$$\begin{array}{c} \frac{\bar{X}}{\bar{Y}}\approx \frac{K_{A}\bar{A}\left(I_{T}-\bar{I}\right)}{K_{I}\bar{I}\left(A_{T}-\bar{A}\right)}=\frac{c_{1}}{c_{2}}, \end{array}$$ (23)

with equality when *β*_4_, *β*_5_ = 0.

The steady-state solution of Eq (19) is given by the Goldbeter-Koshland equation, which depends on $\bar{X}$ and $\bar{Y}$ only through their ratio,

$$\begin{array}{ll} \frac{\bar{F^{*}}}{F_{T}} & =\frac{\left(\frac{\bar{X}}{K_{F}\bar{Y}}-1\right)-\frac{K_{Y}}{F_{T}}\left(\frac{K_{X}}{K_{Y}}+\frac{\bar{X}}{K_{F}\bar{Y}}\right)}{2\left(\frac{\bar{X}}{K_{F}\bar{Y}}-1\right)}\\ & +\frac{\sqrt{{\left[\left(\frac{\bar{X}}{K_{F}\bar{Y}}-1\right)-\frac{K_{Y}}{F_{T}}\left(\frac{K_{X}}{K_{Y}}+\frac{\bar{X}}{K_{F}\bar{Y}}\right)\right]}^{2}+4\frac{K_{Y}}{F_{T}}\left(\frac{\bar{X}}{K_{F}\bar{Y}}-1\right)\left(\frac{\bar{X}}{K_{F}\bar{Y}}\right)}}{2\left(\frac{\bar{X}}{K_{F}\bar{Y}}-1\right)}, \end{array}$$ (24)

where *K*_*F*_ = *β*_6_/*α*_6_.

#### Coupled growth and gradient detection (Fig 4)

We couple the growth and gradient sensing models together as shown in the schematic in Fig 4. Here, the output of the gradient sensor F* modulates the production and transport of the growth substrate *G*, and thus scales neurite growth dependent on whether the growth cone is up- or down-gradient of the cell body. We consider these regions of the cell as two separate well-mixed compartments and explicitly model transport of the activating and inhibitory signals between them as described above. The complete model is described by Eqs (8)–(11), and the following system:

$$\frac{dG}{dt}=\alpha_{0}+\alpha_{F}F^{*}-\alpha_{1}AG+\beta_{1}I_{\text{g}}G^{*}-\beta_{0}G$$ (25)

$$\frac{dG^{*}}{dt}=\alpha_{1}AG-\beta_{1}I_{\text{g}}G^{*}-\beta_{0}G^{*}$$ (26)

$$\frac{dX}{dt}=\alpha_{2}A_{\text{c}}-\beta_{2}X-\gamma_{1}Z_{X}X$$ (27)

$$\frac{dY}{dt}=\alpha_{3}I-\beta_{3}Y-\gamma_{2}Z_{Y}Y$$ (28)

$$\frac{dZ_{X}}{dt}=\alpha_{4}A_{\text{c}}I-\beta_{4}Z_{X}-\gamma_{1}Z_{X}X$$ (29)

$$\frac{dZ_{Y}}{dt}=\alpha_{5}A_{\text{c}}I-\beta_{5}Z_{Y}-\gamma_{2}Z_{Y}Y$$ (30)

$$\frac{dF^{*}}{dt}=\alpha_{6}\frac{X(F_{T}-F^{*})}{K_{X}+\left(F_{T}-F^{*}\right)}-\beta_{6}\frac{YF^{*}}{K_{Y}+F^{*}}.$$ (31)

Analogous to Eq (14), the steady-state growth response is given by

$$\begin{array}{c} \bar{G^{*}}=(k_{0}+\bar{F^{*}}\left(\bar{A},\bar{I}\right))\frac{k_{1}\bar{A}}{k_{2}+k_{3}\bar{A}+\bar{I}}, \end{array}$$ (32)

with *k*_0_ = *α*_0_/*α*_*F*_, $k_{1}=\alpha_{1}\alpha_{F}k_{\text{deg}}^{I}/\beta_{0}\beta_{1}k_{\text{a}}$, $k_{2}=\beta_{0}k_{\text{deg}}^{I}/\beta_{1}k_{\text{a}}$, and $k_{3}=\alpha_{1}k_{\text{deg}}^{I}/\beta_{1}k_{\text{a}}$. The gradient sensor $\bar{F^{*}}$ modulates growth with steady-state determined from Eqs (20), (21) and (24), after replacing $\bar{A}$ with the cell-body signal $\bar{A_{\text{c}}}=\frac{k_{\text{r}}}{k_{\text{deg}}^{A}}\bar{A}$.

For simulations of neurite extension we augment this system with the equation

$$\begin{array}{c} \frac{dR}{dt}=r_{0}+k_{\text{ext}}G^{*}, \end{array}$$ (33)

which describes the increase in neurite length *R* as consisting of a basal rate *r*_0_ and a term proportional to the output of the model G*.

To fit the model to the experimental data (Fig 5A and 5B) we first set the parameters of the mutual-inhibition motif (Eqs (27)–(30)) using order of magnitude estimates, and such that the concentrations of signalling components remained within the range ∼ 1 − 100 nM. Further, we set *α*_4_ = *α*_2_/*I*_*T*_, $\alpha_{5}=\alpha_{3}k_{\text{deg}}^{A}/A_{T}k_{\text{r}}$, and *β*_3_ = *α*_5_*b*_2_*K*_*A*_/*α*_4_*K*_*I*_ to enforce the condition that $\bar{X}/\bar{Y}\approx c_{1}/c_{2}$. With these values fixed, along with those of Eqs (8)–(11) detailed above, we then fitted the parameters of Eqs (25), (26), (31) and (33) through simulations of outgrowth in shallow gradients. Fitting was accomplished by a coarse sweep of parameters to identify combinations that minimised the error between simulated and experimental data, followed by manual refinement by visual inspection. This heuristic approach was efficient because over the long timescale of simulated growth the response is largely determined by the form of the steady-state ${\bar{G}}^{*}$ (Eq (32)), making the role of key parameters clear. Intuitively, for the biphasic outgrowth curve in Fig 5A, the ratio *β*_0_/*α*_1_ and *K*_*I*_ determine the position on the concentration axis of the rising and falling phases, respectively; the ratio *β*_1_/*α*_1_ determines the degree of inhibition at high concentrations; and *r*_0_ in Eq (33) determines the constant offset. Parameter *k*_ext_ serves as a conversion factor between the concentration output of the chemical model (nM) and the neurite growth rate (μm min^−1^), and determines the overall magnitude of the response. The directional bias (Fig 5B) is determined by the ratio *α*_0_/*α*_*F*_, which sets the relative contributions of the growth and gradient sensing motifs, and also by the degree of sensitivity of the enzyme kinetics in Eq (31). In the latter case, the parameters were again selected as order of magnitude estimates, and such that reaction rates and concentrations remained physiological. The parameter values are given in Table 1.

The parameter sensitivities of the model also follow from the form of Eq (32), whether at steady-state, or after a long period of growth in a shallow gradient as in Fig 5A and 5B. For instance, the output depends linearly on *α*_0_, but has a $∼\frac{1}{\beta_{0}\left(\beta_{0}+C\right)}$ dependence on the reverse rate. The model is most sensitive to the parameters that contribute to the output of the gradient sensor $\bar{F^{*}}$ (Eqs (27)–(30)), in which a degree of fine tuning is required to produce the desired processing of the input signals. As in Fig 3D, where we address this issue, small perturbations to these values are well-tolerated, though larger deviations from the equality $\alpha_{3}/\alpha_{5}=A_{T}k_{\text{a}}/k_{\text{deg}}^{A}$, in particular, lead to errors in sensing the direction of the gradient.

### Simulations

Ganglion outgrowth was simulated as a deterministic extension of neurites from the boundary of a disc of radius *R*_*E*_ = 300μm, representing average neurite trajectories in an experimental image plane (cf. Fig 1A). The boundary of the disc was seeded with *N* = 360 model neurites, projecting radially at angles $\theta_{n}=\frac{2\pi n}{N}$. The rate of extension of each neurite was determined from the system of ordinary differential equations for the signalling model (Eqs (25)–(31)) and linear growth rule (Eq (33)). For comparison with the NGF gradient data set, outgrowth was simulated with the explant body disc centred in an exponential gradient, given in polar coordinates by *c*(*r*, *θ*) = *c*_0_ exp(*sr* sin(*θ*)). Here, *c*_0_ denotes the background concentration and *s* denotes the gradient steepness (a 0.1% per 10 μm gradient corresponds to *s* = 1 × 10^−4^). The growth-cone NGF concentration *c*_1_ and cell-body concentration *c*_2_, which drive the signalling model, were determined for each neurite dependent on its length and angle of projection. For a neurite of length *R* at angle *θ*, the cell-body concentration remained fixed at *c*_2_ = *c*(*R*_*E*_, *θ*), whereas the growth-cone concentration was updated as the neurite extended as *c*_1_(*t*) = *c*(*R*_*R*_ + *R*(*t*), *θ*). The model was initialised at steady-state values, and 48h outgrowth was simulated. The results were analysed using the Fourier decomposition described above. All simulations were performed in MATLAB (Mathworks) with custom written code and the solver *ode15s*.

## Supporting information

S1 Fig Quantification of explant and dissociated-cell growth after 96 h. (A) After 96 h, average explant outgrowth was significantly less at 10 nM compared to 0.3 nM NGF. The data points on the left are replotted from the 48 h growth experiment (Fig 4C). (B) Consistent with the results in Fig 4F, no growth inhibition of dissociated cells was observed after 96 h (*p* = 0.81, Mann-Whitney U-test for difference between 0.3 nM and 10 nM conditions, *n* = 91 cells and *n* = 113 cells for 0.3 nM and 10 nM NGF respectively). Within concentration conditions, neurites were longer after 96 h growth compared to 48 h, as expected, confirming a difference can be detected when it is present (*p* = 0.02 and *p* = 4.7 × 10^−5^ for 0.3 nM and 10 nM respectively, Mann-Whitney U-test). These results were robust to the removal of outliers (red crosses).
(EPS)

S2 Fig Examples of image segmentation and positive control samples for quantification of fasciculation. (A) The Ridge Detection algorithm reliably detects neurites (red lines) and estimates widths by averaging the distance between edges (green lines) along each detected segment. Parameters: *σ* = 1.37, lower threshold = 0, upper threshold = 10.03. (B,C) Collections of thin and thick samples (15 patches each) were used as a positive control to test the ability of the algorithm to discriminate distributions of bundle widths.
(EPS)

S3 Fig Examples of image processing for quantification of explant outgrowth. (A,B) Raw images. (C,D) Images are thresholded by pixel intensity into binary masks, and the explant body region removed. (E,F) Boundary curves are fitted to the inner and outer boundary of the neurite region (black). The distance between the two curves, parameterised by angular variable *θ*, defines the radial outgrowth function *R*(*θ*). Fourier approximation of *R*(*θ*) gives a low dimensional representation of explant shape. Shown in E and F are the reconstructions with 1 coefficient (blue), 5 coefficients (green) and 101 coefficients (red). Using 5 coefficients was sufficient to capture the major outgrowth features across all data.
(EPS)

S1 Table Statistical analysis of guidance (comparison to 1 nM plateau with Mann-Whitney U test). (PDF)

S2 Table Pearson correlation between shape properties of outgrowth and explant body. (PDF)
