# Supplementary material for: Control of neurite growth and guidance by an inhibitory cell-body signal
Source: PLoS Comput Biol. 2018 Jun 21;14(6):e1006218. doi: 10.1371/journal.pcbi.1006218 (PMC6013027; doi:10.1371/journal.pcbi.1006218)
Supplement: S1 Table — (PDF) [file pcbi.1006218.s004.pdf]

plateau control										
[NGF] (nM)	1									
$\overline{b_1/a_0}$	-0.02									
0.12% gradient										
[NGF] (nM)	0.0009	0.0092	0.0276	0.092	0.276	0.92	2.76	9.2	27.6	92
$\overline{b_1/a_0}$	-0.03	0.02	0.07	0.10	0.10	0.06	-0.03	6×10^{-4}	-0.02	-0.03
p-value	0.40	0.09	2×10^{-6}	5×10^{-11}	4×10^{-12}	4×10^{-6}	0.60	0.49	0.65	0.60
0.18% gradient										
[NGF] (nM)	0.0011	0.011	0.033	0.11	0.33	1.1	3.3	11	33	110
$\overline{b_1/a_0}$	n/a	0.01	0.05	0.08	0.14	0.05	0.03	-0.08	-0.04	-0.03
p-value	n/a	0.10	3×10^{-4}	3×10^{-7}	4×10^{-14}	7×10^{-5}	6×10^{-3}	3×10^{-3}	0.43	0.53
0.24% gradient										
[NGF] (nM)	0.0015	0.015	0.045	0.15	0.45	1.5	4.5	15	45	150
$\overline{b_1/a_0}$	0.03	0.06	0.08	0.10	0.08	0.03	-0.02	-0.05	-0.03	0.00
p-value	0.03	2×10^{-5}	2×10^{-8}	5×10^{-10}	1×10^{-9}	6×10^{-3}	0.93	0.04	0.28	0.24
0.3% gradient										
[NGF] (nM)	0.0021	0.021	0.063	0.21	0.63	2.1	6.3	21	63	210
$\overline{b_1/a_0}$	0.07	0.08	0.14	0.11	0.04	-0.01	-4×10^{-3}	-0.03	-0.02	-0.05
p-value	2×10^{-5}	3×10^{-6}	4×10^{-14}	9×10^{-12}	9×10^{-3}	0.65	0.21	0.44	0.50	0.19

S1 Table. Statistical analysis of guidance (comparison to 1 nM plateau with Mann-Whitney U test.)
