# Supplementary material for: Control of neurite growth and guidance by an inhibitory cell-body signal
Source: PLoS Comput Biol. 2018 Jun 21;14(6):e1006218. doi: 10.1371/journal.pcbi.1006218 (PMC6013027; doi:10.1371/journal.pcbi.1006218)
Supplement: S2 Table — (PDF) [file pcbi.1006218.s005.pdf]

	a_0	a_1/a_0	b_1/a_0	a_2/a_0	b_2/a_0	a_0^E	a_1^E/a_0^E	b_1^E/a_0^E
a_0	1	-3×10^{-3}	0.15	0.02	0.01	0.17	3×10^{-3}	-0.02
a_1/a_0		1	0.03	4×10^{-4}	1×10^{-3}	-4×10^{-4}	-0.08	-0.02
b_1/a_0			1	0.01	-0.04	-0.07	-0.01	-0.10
a_2/a_0				1	0.04	2×10^{-3}	-7×10^{-3}	0.02
b_2/a_0					1	0.03	0.01	-0.01
a_0^E						1	1×10^{-4}	-0.01
a_1^E/a_0^E							1	5×10^{-3}
b_1^E/a_0^E								1

S2 Table. Pearson correlation between shape properties of outgrowth and explant body.
